# Die Neuropathologin und Hirnforscherin Lieselotte Gerhard (1925–2010)

**DOI:** 10.1007/s00292-026-01545-y

**Published:** 2026-02-19

**Authors:** Ann Britt Siemer, Holger Steinberg

**Affiliations:** Forschungsstelle für die Geschichte der Psychiatrie, Klinik und Poliklinik für Psychiatrie und Psychotherapie, Medizinische Fakultät der Universität Leipzig, Semmelweisstraße 10, 04103 Leipzig, Deutschland

**Keywords:** Geschichte der Frauen in der Medizin, Demenzforschung, Entwicklung der Neuropathologie, Neuroanatomie des Kaninchens, Biografie, History of women in medicine, Dementia research, Development of neuropathology, Neuroanatomy of rabbits, Biography

## Abstract

**Hintergrund:**

Die Biografien und Leistungen der ersten Professorinnen der Medizin in Deutschland sind zum gegenwärtigen Zeitpunkt kaum erforscht. In den Fächern Pathologie und Neuropathologie war Lieselotte Gerhard eine der Ersten. Ziel dieser Arbeit ist es, einen Teil dieser Forschungslücke zu schließen.

**Material und Methoden:**

Die Studie geht auf das Sammelwerk von Boedeker und Meyer-Plath zurück, welches Lieselotte Gerhard als eine der ersten Habilitandinnen in Deutschland aufzählt. Auf Grundlage der Publikationen von Gerhard, Archivquellen, Sekundärliteratur sowie Interviews wurde ihre wissenschaftliche Tätigkeit und Biografie rekonstruiert und kontextualisiert.

**Ergebnisse:**

Die Biografie der Forscherin offenbart einen für ihre Zeit außergewöhnlichen Lebensweg. Gerhard begann bei den bedeutenden Hirnforschern Oskar und Cécile Vogt zu lernen und durchlief viele, auch internationale angesehene Stationen. Schließlich baute sie ihr eigenes Institut als erste deutsche Professorin der Neuropathologie an der Gesamthochschule Essen auf. Insgesamt zeichnete sich ihre Forschung durch die enge Verbindung klinischer und pathologischer Perspektiven aus. Mit ihrer Habilitationsschrift gelang ihr die Herausgabe eines zeitgenössischen Standardwerks.

**Diskussion:**

In der Zusammenschau wird deutlich, dass die Forscherin Gerhard eine signifikante Leistung für die Entwicklung der Neuropathologie erbrachte. Außerdem gelang es ihr, bestehende geschlechterspezifische Barrieren durch ihre Zielstrebigkeit und Durchsetzungskraft zu durchbrechen. Ihre Beiträge verdienen daher eine erneute Aufmerksamkeit und Würdigung.

**Zusatzmaterial online:**

Zusätzliche Informationen sind in der Online-Version dieses Artikels (10.1007/s00292-026-01545-y) enthalten.

## Abstract (en)

**Background:**

To date, little research has been conducted on the lives and contributions of the first women appointed as professors of medicine in Germany. Lieselotte Gerhard was one of these first female professors in the fields of pathology and neuropathology. The aim of this study is to address this research gap.

**Materials and methods:**

This study draws on Boedeker and Meyer-Plath’s compilation of Germany’s first female habilitation candidates, in which Lieselotte Gerhard appears under pathology and neuropathology. Based on Gerhard’s publications, as well as archival sources, secondary literature, and interviews, her research work and biography were reconstructed and contextualised.

**Results:**

The researcher’s biography reveals a remarkable life path for her time. Gerhard began her studies with the distinguished brain researchers Oskar and Cécile Vogt and held many esteemed positions, including international ones. Finally, she established her own institute at the University of Essen as Germany’s first female professor of neuropathology. Overall, her research was characterized by a close integration of clinical and pathological perspectives. With her habilitation thesis, she succeeded in publishing a contemporary standard reference work.

**Discussion:**

Overall, Gerhard made a significant contribution to the development of her field. Through her determination and perseverance she was able to challenge and overcome existing gender barriers. Her achievements therefore merit renewed attention and presentation.

Über die wissenschaftlichen Leistungen von Frauen in der Geschichte der Neurowissenschaften in Deutschland ist wenig bekannt, deren Beitrag für die Entwicklung dieser Fachdisziplinen wenig erforscht und insgesamt deswegen wenig gewürdigt. Eine dieser weitgehend unbekannten Medizinerinnen war Lieselotte Gerhard. Sie gehört zum Kreis der ersten 15 Frauen in Deutschland, die auf dem Gebiet der Psycho- und Neurowissenschaften habilitierten [3], und erhielt als erste Frau eine ordentliche Professur in der Neuropathologie. Die vorliegende Untersuchung möchte ihr Werk vor dem Hintergrund der männerdominierten Strukturen der medizinischen Hochschulen vorstellen, einordnen und konkret nachfragen, welchen Beitrag zur Forschung sie leistete.

## Methodik

Ausgangspunkt der Forschung ist eine Übersichtsarbeit von Boedeker und Meyer-Plath zu den ersten Habilitandinnen in Deutschland, die Lieselotte Gerhard als Hirnforscherin auflistet [3]. Diese Anregung aufnehmend basiert unsere Studie auf folgend eruierten Publikationen von Lieselotte Gerhard, Archivalien aus umfangreichen Personalakten ihrer verschiedenen universitären Anstellungen und ergänzender Primär- und Sekundärliteratur zur Einordnung der wissenschaftlichen Erkenntnisse und biografischen Gegebenheiten. Zunächst wurde im Mai 2023 der aktuelle Forschungsstand zu der Person durch wiederholtes Suchen nach dem Namen der Forscherin in Katalogen des Karlsruher Virtuellen Katalogs (KVK), der Deutschen Nationalbibliothek und der Universitätsbibliothek Leipzig erfasst. Die Ergebnisse beschränkten sich auf die Publikationen von Gerhard selbst. Eine wissenschaftliche Erforschung der Biografie und des Werks von Gerhard hat nach diesen Erkenntnissen bisher nicht stattgefunden. Folglich wurden zunächst für eine Rekonstruktion der Biografie – basierend auf Hinweisen in Nachrufen sowie anhand der Erscheinungsjahre und Institutsangaben ihrer Publikationen – Nachforschungen in den jeweiligen Universitätsarchiven durchgeführt. So wurden beispielsweise selbstverfasste Lebensläufe und eine Liste von wissenschaftlichen Arbeiten, die die Forscherin für ihre Habilitation 1962 in Düsseldorf und ihre Bewerbung um die Professur in Essen 1972 einreichte, gefunden [17, 30]. Im August 2025 wurde durch Eingabe des Namens der Autorin und der Titel ihrer Publikationen gezielt in Datenbanken (KVK, PubMed und Web of Science) und in Katalogen der Universitätsbibliothek Leipzig und der Deutsche Nationalbibliothek gesucht. Neben diesen schriftlichen Quellen wurde auch Oral History zum weiteren Informationsgewinn genutzt. Dabei basiert die konkrete Vorgehensweise auf den Richtlinien des Historikers Donald A. Ritchie [23]. Zum einen wurde ein persönliches Interview vor allem für biografische und charakterliche Beschreibungen mit Heinz Ziegler, dem Mann der Cousine der Forscherin und langjährigem Freund, geführt. Zur weiteren Ergänzung der fachlichen Perspektive wurde außerdem der Pathologe Wolfgang Feiden zu seiner ehemaligen Doktormutter telefonisch befragt.

## Zur Biografie

**Abb. 1:**
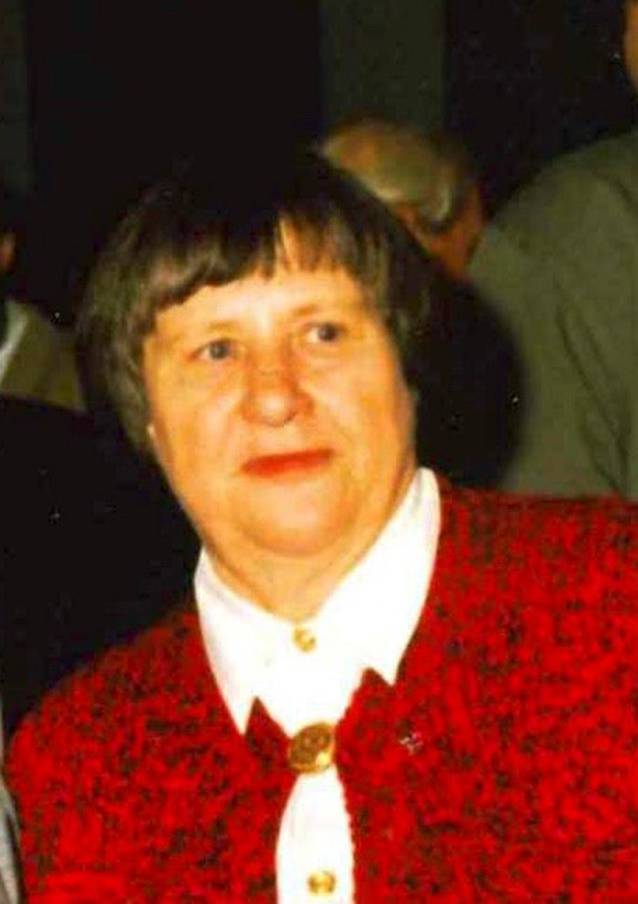
Lieselotte Gerhard. Bildquelle: Heinz Ziegler, Leipzig (Privatbesitz)

Lieselotte Gerhard (Abb. 1) wurde am 24.08.1925 in Potsdam geboren. In ihrer Schulzeit zog die Familie mehrfach um, ihr Abitur legte sie dann 1944 in der Eifel ab. Darauf folgte der zu dieser Zeit verpflichtende Arbeitsdienst. Im Jahr 1946 kam Gerhard durch ein einjähriges Praktikum in dem angesehenen Institut für Hirnforschung in Neustadt im Schwarzwald zum ersten Mal mit Hirnforschung in Berührung. Dieses private Institut war von Cécile und Oskar Vogt 1937 gegründet worden, nachdem Oskar Vogt im selben Jahr von den nationalsozialistischen Machthabern aus seiner Position als Leiter des Kaiser-Wilhelm-Instituts für Hirnforschung in Berlin entlassen worden war [31]. Das Institut befand sich in der Nähe des Bauernhofs, wo Gerhard den Arbeitsdienst ableistete [17, Lebenslauf Gerhard, 5]. Es kann angenommen werden, dass sie hier die maßgebliche Anregung für ihr eigenes Forschungsfeld fand. Später wurde Gerhard dementsprechend in der Aufteilung der „Schulen der Nervenheilkunde“ zu der Berliner Schule um Oskar Vogt gezählt [21]. Sie studierte Medizin von 1947 an zunächst in Tübingen und von 1950 bis zu ihrem Abschluss 1953 in Düsseldorf [30, Lebenslauf]. Nach dem Staatsexamen arbeitete sie erneut bei den Vogts. Diese Forschungen bildeten die Grundlage für ihre Promotion an der Medizinischen Akademie Düsseldorf über Kleinhirnveränderungen bei „amaurotischer Idiotie“ [6, 30, Lebenslauf]. Diese Erkrankung ist heute unter den Namen Morbus Tay-Sachs oder GM2-Gangliosidose Typ 1 bekannt. Gerhard war in den folgenden Jahren für mehrere Forschungsaufenthalte in Kanada und in den USA. Für über 2 Jahre forschte sie bei dem damals bekannten Hirnforscher Wilder Penfield am Montreal Neurological Institute. Diese Zeit habe ihre Forschungsausrichtung von einem rein neuroanatomischen um einen klinisch-pathologischen Ansatz erweitert [30, Brief Meessen].

Gerhard begann 1957 am Pathologischen Institut der Medizinischen Akademie in Düsseldorf zu arbeiten und durchlief dort in den folgenden Jahren mehrere Positionen. Sie begann als wissenschaftliche Assistentin, stieg zur Kustodin auf und erhielt schließlich die Positionen einer außerplanmäßige Professorin im Jahr 1969 und die der wissenschaftlichen Rätin nur ein Jahr später [30]. Während dieses Zeitraums habilitierte sie sich im Jahr 1962 im Fach Allgemeine Pathologie und Neuropathologie mit der Monografie *Atlas des Mittel- und Zwischenhirns des Kaninchens* [7]. Im Zuge des Habilitationsverfahrens wurden auch Gerhards Stärken abseits des Fachlichen von Hubert Meessen (zu dem Zeitpunkt Leiter des Pathologischen Instituts Düsseldorf) gelobt, sie sei „eine menschlich so geschlossene und gefestigte Persönlichkeit“ und habe darüber hinaus eine „große Anerkennung“ für ihre Lehrtätigkeit verdient [30, Brief Meessen].

Nach 16 Jahren wissenschaftlicher Tätigkeit in Düsseldorf folgte 1973 die Berufung an die Gesamthochschule Essen (aus dieser Hochschule wurde durch die Fusion mit der Universität Duisburg im Jahr 2003 die Universität Duisburg-Essen), wo sie im selben Jahr die Professur für Neuropathologie übernahm [25]. In der bundesdeutschen Hochschullandschaft der 1970er-Jahre erfolgte eine deutliche Erweiterung, die sich in zahlreichen Neugründungen von Hochschulen und der Vergrößerung der bestehenden Einrichtungen zeigte. Dies könnte eine deutliche Zunahme der Professuren für Frauen vermuten lassen, was allerdings nur in einem geringen Maß geschah [16]. Weiblich besetzte ordentliche Professuren blieben die völlige Ausnahme. Während im Jahr 1966 der Anteil an weiblichen Professuren in der BRD 2,2 % betrug, stieg dieser Wert bis 1977 lediglich auf 3,8 % [28]. An den Medizinischen Fakultäten in Westdeutschland gab es im Jahr 1966 beispielsweise nur 6 Lehrstuhlinhaberinnen [16]. Gerhard wurde durch ihre Berufung die erste Frau auf einem Lehrstuhl für Neuropathologie [5]. Zur Einordnung wird darauf verwiesen, dass in der eng verwandten Disziplin Pathologie die erste Forscherin im Jahr 2003 berufen wurde [14]. Nach wenigen Jahren war das neugegründete Institut unter der Leitung von Gerhard so anerkannt, dass beispielsweise im Jahr 1979 die Deutsche Gesellschaft für Neuropathologie und Neuroanatomie (DGNN) in Essen für ihre jährliche Tagung zusammenkam [20]. Das Institut zeichnete sich in der wissenschaftlichen Arbeit durch einen starken Bezug zur Klinik aus. Es wurden nicht nur einzelne Präparate für die Beurteilung herangezogen, sondern komplette Sektionen und Betrachtungen aller Befunde waren für die abschließenden Beurteilungen entscheidend [5]. Darüber hinaus setzte sich Gerhard besonders für die Lehre ein und blieb trotz ihrer Emeritierung im Jahr 1993 bis ins Wintersemester 2002/03 im Lehrbetrieb aktiv [29]. Außerdem arbeitete sie noch viele Jahre lang an einem an die Klinik Holthausen angegliederten Institut für klinische Neurochirurgie von Werner Ischebeck in Hattingen und nahm weiterhin am wissenschaftlichen Diskurs teil.^1^ Lieselotte Gerhard verstarb am 17.02.2010.

## Zur Forschung

Im Rahmen der Recherchen wurde ein Werkverzeichnis Lieselotte Gerhards erstellt. Es umfasst insgesamt 117 Titel, sowohl Monografien und Buchkapitel als auch Aufsätze in wissenschaftlichen Zeitschriften (s. Supplement 1). Die Arbeit der Forscherin zeigt eine große thematische Breite und umfasst einen Publikationszeitraum von fast 50 Jahren zwischen 1956 und 2005. Sich häufende Themen sind Tumorerkrankungen in Gehirn und Rückenmark, Enzephalitiden und neurodegenerative Erkrankungen wie dementielle Erkrankungen und die Parkinson-Krankheit. Daneben finden sich auch spezifische Themen, die meist in 2 bis 3 aufeinanderfolgenden Arbeiten und Vorträge behandelt wurden. Beispiele dafür sind die generalisierte Blastomykose [11] und die Schädigung des Rückenmarks nach Starkstromunfällen [9]. Bei vielen dieser Erkrankungen steht ein klinisch-pathologischer Ansatz im Vordergrund. Wegen der Anzahl und der thematischen Vielfalt der Forschung Gerhards werden in diesem Artikel schlaglichtartig 2 ihrer zentralen Themen näher analysiert.

### Habilitation *Das Mittel- und Zwischenhirn des Kaninchens*

Der *Atlas des Mittel- und Zwischenhirns des Kaninchens* von Lieselotte Gerhard erschien im Jahr 1968 im Springer-Verlag. Er stellt die publizierte Fassung ihrer bereits 1962 eingereichten Habilitationsarbeit dar [17, Lebenslauf]. Dabei handelt es sich um eine Fortsetzung und Ergänzung der Arbeiten ihres Vorgesetzten Meessen und des polnischen Neurologen, Hirnforschers und Vogt-Schülers Jerzy Olszewski, vor allem deren Untersuchung des Rautenhirns des Kaninchens aus dem Jahr 1949 [18]. Die 3 Neurowissenschaftler strebten gemeinsam an, das zuvor genutzte Standardwerk von Cornelius Winkler und Ada Potter aus dem Jahr 1911 [32] zu ersetzen. Insbesondere wollten sie durch die Abbildung mikroskopischer Übersichtsaufnahmen von Originalschnitten eine objektivere und reproduzierbare Information für experimentelle Untersuchungen bieten [7]. Das Werk von Winkler und Potter weist keine Fotografien, sondern nur Zeichnungen auf, was die Gefahr einer eingeschränkten Objektivität birgt. Gerhards Atlas entspricht dem Umfang eines klassischen Anatomieatlas und beschäftigt sich mit den Hirnstrukturen des Kaninchens. Der erste Teil des Atlas stellt durch makroskopische Fotografien zunächst eine Übersicht über die genutzten Präparate dar. Diese Fotografien sind durch detaillierte schematische Darstellungen und Röntgenaufnahmen der einzelnen Teile mit Beschriftungen der neuroanatomischen Strukturen ergänzt. Es folgt eine genaue Übersicht zur Schnittführung bei diesen Präparaten, sodass diese dann im ausführlichen Hauptteil des Atlas, den mikroskopischen Aufnahmen, genau nachzuvollziehen sind. Auch in diesem Abschnitt des Atlas sind die Aufnahmen beschriftet und durch präzise Beschreibungen auf Deutsch und Englisch ergänzt. Im abschließenden Teil findet sich eine Diskussion einzelner neuroanatomischer Strukturen, dabei wird auch auf die Ergebnisse anderer Forschungsarbeiten Bezug genommen [7]. Meessen stellt in seinem Gutachten über die Habilitationsschrift besonders heraus, dass es Gerhard gelungen sei, die untersuchten Kerngebiete in verschiedenen Schnittebenen in Bezug aufeinander darzustellen. Dies sei in seinem eigenen Atlas noch nicht gelungen. Außerdem sei die Darstellung von Bezügen zwischen den einzelnen anatomischen Strukturen außergewöhnlich gut. Diese systematische Untersuchung ist nach Meessen ein besonderer Gewinn für zukünftige Forschung [30]. Die Bewertung wird von den Korreferenten, den damaligen Professoren für Anatomie und Neurologie an der Medizinischen Akademie in Düsseldorf, Anton Kisselbach und Eberhard Bay, geteilt. Beide betonten darüber hinaus, dass der Atlas von „dann an“ das Standardwerk in der Forschung darstelle. Die grundlegende Arbeit Gerhards sei vor allem für die vergleichenden physiologisch-morphologischen Untersuchungen von besonderer Relevanz [30]. Die Forscherin beschäftigte sich auch mit verwandten Themen. Bereits 1 Jahr nach dem Erscheinen ihres Atlas veröffentlichte sie gemeinsam mit Olszewski einen Band über die Medulla oblongata und den Pons als Teil des Handbuchs der Primatenkunde *Primatologia*. In diesem Band werden eigene Forschungen zu den neuroanatomischen Strukturen unterschiedlicher Primaten präsentiert [12].

### Demenzforschung

Ein Forschungsschwerpunkt Gerhards in den 1960er-Jahren liegt im Bereich der demenziellen Erkrankungen. So beschäftigt sich Gerhard gemeinsam mit ihrer Düsseldorfer neurowissenschaftlichen Kollegin Elfriede Albert in einer Publikation mit der Auswertung von pathologischen Befunden von Patienten, die an der „senilen Demenz“ und der „Alzheimerschen Krankheit“ litten. Dabei werden die jeweiligen Krankengeschichten mit detaillierten Angaben zu klinischen Befunden dargestellt und um entsprechende ausführliche pathologische Befunde ergänzt. In diesen Untersuchungen wird von den Forscherinnen ein Zusammenhang zwischen der Ausprägung der Hirnpathologie und dem Ausmaß der Klinik festgestellt. Allerdings sei eine Differentialdiagnose beider Erkrankungen ausschließlich durch histologische Untersuchungen entsprechend dem damaligen Forschungsstand – auch in dieser Studie – nicht möglich gewesen. Nur die Zusammenschau von Klinik und Pathologie führe zu der Diagnose [1]. Gerhard lässt eine methodisch ähnliche Arbeit folgen, in der dieses Mal eine klinisch-pathologische Betrachtung der Diagnose „Cerebralsklerose“ in Abgrenzung zur „senilen Demenz“ durchgeführt wird [8]. Die historische Bezeichnung der „Cerebralsklerose“ lässt sich nach heutigem Verständnis am ehesten der vaskulären Demenz zuordnen. Beide Arbeiten sind mit mehreren makroskopischen und mikroskopischen Bildern versehen.

In den 1970er-Jahren beschäftigt sich Gerhard anhand vergleichender Fallstudien unter anderem mit der These, dass bei der „Alzheimerschen Krankheit“ eine genetische Komponente mitwirke. In einer Publikation aus dem Jahr 1972 strebt sie eine Abgrenzung der atypischen „Alzheimerschen Krankheit“ von der typischen „Alzheimerschen Krankheit“ an. Anders als im heutigen Verständnis steht hier die atypische Variante für Fälle mit besonders frühem Erkrankungsbeginn [10]. Entsprechend dem zeitgenössischen Forschungsstand ist die Klassifikation der dementiellen Erkrankungen insgesamt uneinheitlich und teilweise widersprüchlich; die Begriffe der „senilen Demenz“, der „Alzheimerschen Krankheit“ und des „arteriosklerotischen Irrseins“ lassen sich in neuropsychiatrischen Hand- und Lehrbüchern in unterschiedlichen Definitionen und Abgrenzungen voneinander finden [2, 24]. Diese Unschärfe der Begriffe lässt sich auch in der historischen Entwicklung der Klassifikationssysteme erkennen. Die ICD‑7, 1958 in der deutschen Fassung herausgegeben, und die 10 Jahre später folgende ICD‑8 differenzieren lediglich zwischen „präseniler“ und „seniler Demenz“ [26, 27]. Eine Abwendung von dieser Einteilung erfolgt erst in der ICD-10. Dass das Verständnis dieser Erkrankungen und damit ihre Benennung und Klassifizierung auch heute noch nicht abgeschlossen sind, wird in den Neuerungen von ICD-10 zu ICD-11 aktuell wieder deutlich [15].

In den 1980er-Jahren ist in der Publikationsaktivität Gerhards eine Veränderung des Schwerpunkts festzustellen. Sie beschäftigt sich unter anderem mit weiteren degenerativen Erkrankungen und Traumata des Zentralnervensystems. In dieser Phase bleibt der enge klinische Bezug ihrer neuropathologischen Arbeiten ein durchgängiges Motiv ihrer Forschung. In den Jahren 1992 und 2002 wirkt Gerhard zudem durch ihre pathologischen Präparate an weiteren Publikationen im Bereich der Demenzforschung mit [4, 19].

## Forschung, Karriere und Geschlecht im Kontext ihrer Zeit

Lieselotte Gerhard fand, was wahrscheinlich eher ungewöhnlich ist, zuerst ihre Leidenschaft für ein spezielles Fachgebiet der Medizin und begann dann mit dem Medizinstudium [17]. Über ihre gesamte wissenschaftliche Karriere hinweg ist deutlich die Leidenschaft für die Pathologie und insbesondere für die Neuropathologie zu erkennen. Innerhalb dieses medizinischen Fachgebietes zeigt sich ein vielfältiges Interesse an den unterschiedlichsten Fragestellungen. Aus der erstellten Publikationsliste geht hervor, dass die Forscherin in ihrer fast 50-jährigen Publikationstätigkeit Studien zu einer großen Breite von neuropathologischen, aber vereinzelt auch pathologischen Themen veröffentlichte. Mehrfach wurde herausgehoben, dass ihre Arbeiten sich durch eine enge Verzahnung pathologischer Diagnostik mit dem jeweiligen klinischen Bild auszeichneten. Der *Atlas des Mittel- und Zwischengehirns des Kaninchens* stellt einen zentralen Teil ihrer Forschung dar. Einerseits wegen der schlichten Tatsache, dass dieser die Habilitation ermöglichte. Andererseits ist die Bedeutung des Atlas durch die Wertschätzung anderer Forscher – und dies nicht nur im Rahmen des Berufungsverfahrens an die Gesamthochschule Essen – belegt. In einer Buchrezension wird der Beitrag gewürdigt: „It is beyond any doubt that this atlas will remain, for a long time, the standard reference book on this topic“ [22]. Somit ist festzuhalten, dass Gerhard in einem spezifischen Bereich der Forschung mit ihrem Atlas einen großen Einfluss gehabt hat und dabei, genau wie es ihr selbst formuliertes Ziel war, eine Forschungslücke der Zeit geschlossen hat [22].

Ein vergleichbarer durchgreifender Erfolg ist bei den hier untersuchten Beiträgen der Wissenschaftlerin zur Demenzforschung nicht unmittelbar feststellbar. Obwohl das Thema von ihr mehrfach aufgegriffen wurde, ordnen sich die Beiträge der Forscherin insgesamt in den bestehenden Forschungsstand ein. Vielleicht lässt sich an dieser Stelle auch eine gewisse Schwäche in Gerhards Forschungsstrategie erkennen: Die große thematische Vielfalt ihrer Arbeit mag einerseits Ausdruck ihres umfassenden Interesses gewesen sein, könnte aber andererseits eine tiefere Spezialisierung behindert haben. Betrachtet man ihr Vorgehen vor dem Hintergrund der Entwicklung ihres Faches, erscheint dies nachvollziehbar. Die Neuropathologie war zwar von einer zunehmenden Institutionalisierung geprägt – Beispiele für diese Entwicklung des Faches sind die Entstehung einer eigenen neuropathologischen Vereinigung 1950 und die Möglichkeit einer separaten fachärztlichen Weiterbildung 1987 [13] –, könnte aber als neues Fach weniger klar ausdifferenzierte Forschungsschwerpunkte als andere lang etablierte Fächer gehabt haben. Vielleicht meinte Gerhard, dass sie in dieser Phase der Entwicklung ihres Faches als Lehrstuhlinhaberin den Gesamtüberblick bewahren sollte. Eine erschöpfende Analyse der wissenschaftlichen Leistung unter Berücksichtigung aller Forschungsthemen und deren Bedeutung muss aufgrund des Umfangs und der Breite der Forschung von Gerhard einer weiterführenden Aufarbeitung vorbehalten bleiben.

Dennoch ist festzuhalten, dass die Gesamtheit der Forschung in den unterschiedlichen Bereichen und über eine lange Zeitspanne hinweg definitiv ein weit überdurchschnittliches wissenschaftliches Lebenswerk darstellt. Gerhard wurde an einen neu geschaffenen Lehrstuhl für Neuropathologie berufen. Der Aufbau und die Etablierung dieses neuen Instituts sind als weiteres Verdienst der Forscherin anzusehen. Ihre engagierte und lang andauernde Lehrtätigkeit ist ebenfalls hervorzuheben. Auch die Betreuung zahlreicher Doktorarbeiten^2^ ist ein Hinweis auf dieses Engagement. Gerhard brachte sich in mehreren Fachgesellschaften intensiv ein [5]. In der Publikationsliste wird ferner die internationale Ausrichtung ihrer Arbeit ersichtlich. Sie publizierte mit Wissenschaftlern unter anderem aus Montreal, Peking und Helsinki in deutschen und internationalen Zeitschriften. Sie war ganz offensichtlich international vernetzt, diese Orientierung könnte nicht zuletzt mit ihren Forschungsaufenthalten als junge Ärztin in Kanada und den USA zusammenhängen. Für die klinische Ausrichtung ihrer neuropathologischen Forschungen erstellte Gerhard Sammlungen von Präparaten mit den entsprechenden Krankengeschichten. Ihr Doktorand Wolfgang Feiden erinnert an die beträchtliche Menge an Materialien, die seiner Doktormutter nach ihrer Berufung von Düsseldorf nach Essen folgten. Im Essener Institut sei eine große Sammlung von Präparaten kompletter Sektionen mit der entsprechenden Krankengeschichte gesammelt worden: „Die ganze Thematik dessen, was heute unter ‚brain banking‘ subsumiert wird, hat Gerhard damals schon im Blick gehabt“ [5]. Der Nutzen dieser Sammlung zeigt sich auch in ihren Publikationen, denn selbst noch als über 75-jährige Forscherin greift Gerhard darauf zurück.

In der Geschichte der deutschen Neuropathologie wurde, abgesehen von Cécile Vogt, bislang keine weitere Wissenschaftlerin eingehend berücksichtigt oder erforscht. Vor dem Hintergrund der historischen Realität der männlich dominierten Forschung erscheint dies nicht ungewöhnlich. Um die Leistung einer Forscherin zu verstehen, ist dieser Kontext allerdings relevant. Frauen waren in der medizinischen Wissenschaft in höheren Positionen eine absolute Ausnahme. Die Koautoren von Gerhards Publikationen waren bis auf sehr wenige Ausnahmen Männer, die Konkurrenz in ihrem Berufungsverfahren waren Männer und in jedem, das Verfahren betreffenden Dokument in Gerhards Personalakten ist deutlich spürbar, dass eigentlich keine Frau für die Position vorgesehen war. Dies könnte das Mindset einer Pionierin erwarten lassen, also einer Frau, die für spätere Generationen Wege öffnen wollte. Ein Hinweis darauf, dass Gerhard sich selbst in dieser Rolle als Wegbereiterin sah, ist jedoch nicht greifbar. Sie hat zwar die besonderen Herausforderungen und Hürden, die sie als Frau in ihrem Beruf meistern musste, benannt, daraus hat sich aber kein Bestreben ergeben, Frauen besonders zu fördern und diese Ungleichheit zu verringern. Dem persönlichen Eindruck des langjährigen Freundes nach habe sie immer das Fachliche im Vordergrund gesehen und so auch keine geschlechtsspezifischen Unterschiede, z. B. unter ihren Doktoranden, gemacht.^3^ Dass Gerhard in der damaligen Zeit eine akademische Karriere gelang, könnte durch 2 Umstände erleichtert worden sein. Sie wurde während der Expansionsbewegung der Hochschulen auf einen Lehrstuhl berufen. Eine Situation, in der zusätzliche Positionen zu besetzen waren, machte die Ernennung einer Wissenschaftlerin wahrscheinlicher, wenn auch die zu erwartende deutliche Gesamtzunahme an weiblich besetzten Stellen nicht stattfand. Förderlich könnte auch die parallel zu Gerhards Berufsleben stattfindende Institutionalisierung des Faches Neuropathologie gewesen sein. In einem neu entstandenen Hochschulfach scheint es möglich, dass hochqualifizierte Frauen, die noch dazu von Begründern und Koryphäen des Faches ausgebildet worden waren, schwerer zu ignorieren waren als anderswo und zu anderen Zeiten.

## Ausblick

Gerhard nahm zu ihrer Zeit eine Ausnahmestellung als Professorin für Neuropathologie ein. Zumindest in der deutschen Neuropathologie und auch in der Hirnforschung arbeitete sie im Feld der Spitzenforscher und trug durch ihre Lehrtätigkeit zur Weiterentwicklung des Faches bei. Als Frau ihrer Generation hat sie einen außergewöhnlichen beruflichen Werdegang aufzuweisen, der ohne ein erhebliches Maß an charakterlicher Belastbarkeit und an Durchhaltevermögen nicht möglich gewesen wäre. Auch wenn die Forscherin es wohl nicht als ihr Ziel benannt hätte, wird sie durch ihre Laufbahn den Weg für nachfolgende Forscherinnen zugänglicher gemacht haben. Die hier vorgestellte Ärztin und Wissenschaftlerin verdient es, nicht vergessen zu werden.

## Fazit für die Praxis

- Die vorliegende Studie zeigt die Neuropathologin Lieselotte Gerhard als eine der Wegbereiterinnen für Frauen in der Medizin, insbesondere in den Neurowissenschaften.
- Ihre Forschung ist geprägt von einer großen thematischen Breite und einer langen Publikationstätigkeit. Sie beschäftigte sich u. a. mit Demenzen. Eine tiefe und besonders längerfristige Spezialisierung als Expertin für einen Bereich ist nicht zu erkennen.
- Die Habilitationsschrift der Forscherin stellt ihren größten einzelnen Forschungserfolg dar. Sie schuf damit eine neuroanatomische Grundlagenarbeit.

## Supplementary Information

ESM1: Publikationsliste Lieselotte Gerhard

## Footnotes

1 Heinz Ziegler, persönliches Interview am 17.06.2024 in Leipzig.

2 Heinz Ziegler, persönliches Interview am 17.06.2024 in Leipzig.

3 Heinz Ziegler, persönliches Interview am 17.06.2024 in Leipzig.
