# Supplementary material for: Neuropathologist and brain researcher Lieselotte Gerhard (1925–2010)
Source: Pathologie (Heidelb). 2026 Feb 19;47(3):214–9. [Article in German] doi: 10.1007/s00292-026-01545-y (PMC13109280; doi:10.1007/s00292-026-01545-y)
Supplement: Supplementary file 1 — ESM1: Publikationsliste Lieselotte Gerhard [file 292_2026_1545_MOESM1_ESM.pdf]

Publikationsliste Liselotte Gerhard; Forschungsstand 29.1.2026

Hinweis: Die Publikationsliste beruht bis einschließlich 1972 auf einem von Lieselotte Gerhard selbst angefertigten Verzeichnis ihrer Arbeiten[1]. Ab 1973 erfolgte eine Ergänzung durch systematische Recherchen in den Datenbanken PubMed und Web of Science.

1. Gerhard L (1956) Gestaltung und Verteilung von Kleinhirnveränderungen bei amaurotischer Idiotie. Journal für Hirnforschung 2:156–224.
2. Gerhard L, Meessen H, Veith G (1958) Normale Anatomie und Pathologie des Nervensystems. In: Cohrs P, Jaffe R, Meessen H (Hrsg) Pathologie der Laboratoriumstiere. Springer, Heidelberg, S 698–720.
3. Gerhard L (1959) Ein Fall von Amyloidose des Gehirns. Zentralblatt für allgemeine Pathologie und pathologische Anatomie 100:282.
4. Gerhard L (1959) Demonstration zu einer neuen kombinierten Darstellung von Markscheiden und Achsenzylindern. Zentralblatt für allgemeine Pathologie und pathologische Anatomie 100:284.
5. Gerhard L, Holländer H (1960) Eine Methode zur kombinierten Darstellung von Markscheiden und Neuriten. Deutsche Zeitschrift für Nervenheilkunde 180:684–687.
6. Gerhard L (1961) Zur Morphologie der perinatalen Hypoxie. In: Zweites Düsseldorfer Symposium: Probleme der ersten Lebenszeit, 25.06.1960. Schattauer, Stuttgart, S 684–687.
7. Gerhard L (1963) Hirn- und Rückenmarksdegeneration bei Bronchial-Carcinom. Zentralblatt für allgemeine Pathologie und pathologische Anatomie 104:212.

1. [1] Landesarchiv Nordrhein-Westfalen, Gesamthochschule Essen. Personalakte. Gerhard. Nr. 11808. Verzeichnis der Arbeiten.

8. Gerhard L (1964) Spätveränderungen des Zentralnervensystems nach Poliomyelitis. Zentralblatt für allgemeine Pathologie und pathologische Anatomie 106:373.

9. Gerhard L, Kockott G (1965) Zur Frage der Häufigkeit und Entstehung der Wernicke'schen Encephalopathie bei schwerer Trunksucht. Klinische Wochenschrift 43:122.

10. Albert E, Gerhard L (1965) Korrelation zwischen klinischen Herdstörungen und pathologisch-anatomischem Befund bei der senilen Demenz und der Alzheimer'schen Krankheit. Fortschritte der Neurologie Psychiatrie und ihrer Grenzgebiete 33:38–43.

11. Gerhard L (1965) Wernicke'sche Erkrankung des Kindesalters und subakute nekrotisierende Encephalopathie. Zentralblatt für allgemeine Pathologie und pathologische Anatomie 107:309.

12. Gerhard L (1966) Diskussionsbemerkung zum Vortrag von H. Bickel Die Prophylaxe von Hirnschäden, verursacht durch Enzymopathien. In: Klent R, Hüter K (Hrsg) Die Prophylaxe frühkindlicher Hirnschäden. Thieme, Stuttgart.

13. Gerhard L, Dönecke K (1966) Morphologische Veränderungen bei generalisierter Blastomykose. Zentralblatt für allgemeine Pathologie und pathologische Anatomie 108:445–446.

14. Gerhard L, Bergener M (1966) Altersgebundene Veränderungen an pigmentierten Nervenzellen des Gehirns unter besonderer Berücksichtigung der submikroskopischen Morphologie. In: Proceedings of the 7th International Congress of Gerontology. Wien, S 213–219.

15. Scheid W, Stammler A, Ackermann R, Gerhard L, Bleifeld W (1966) Encephalitiden bei Herpes-simplex-Virusinfektionen. Fortschritte der Neurologie Psychiatrie und ihrer Grenzgebiete 34:625–647.

16. Gerhard L (1966) Akute Encephalitis oder Encephalopathie bei Tubencarcinom. Zentralblatt für allgemeine Pathologie und pathologische Anatomie 109:445.

17. Gerhard L, Bergener M (1966) Beitrag zum Creutzfeld-Jakob-Syndrom. Zentralblatt für allgemeine Pathologie und pathologische Anatomie 109:300.

18. Gerhard L (1967) Zur vergleichenden Anatomie und Histologie vegetativer Kerngebiete in Mittelhirn, Brücke und Medulla oblongata. Acta Neurovegetativa 30:155–168.

19. Bergener M, Gerhard L (1967) Zur Klinik und Elektroencephalografie des Creutzfeld-Jakob-Syndroms. Archiv für Psychiatrie und Nervenkrankheiten 209:351–364.

20. Gerhard L (1967) Morphologische Veränderungen des Zentralnervensystems bei Schizophrenien. In: Forum der Psychiatrie, Band 19, S 148–156.

21. Gerhard L, Weber H (1967) Encephalitis bei Morbus Behçet. Zentralblatt für allgemeine Pathologie und pathologische Anatomie 110:393–394.

22. Gerhard L (1968) Frühe, neurologische Symptome beim Bronchial-Carcinom. Medizinische Gesellschaft Düsseldorf, 11.01.1967, Klinische Wochenschrift.

23. Weber H, Gerhard L (1968) Chronisch-rezidivierende Leukoencephalitis bei Morbus Behçet. Zentralblatt für die gesamte Neurologie und Psychiatrie 192:114.

24. Gerhard L, Bergener M, Reinhardt V, Eichenauer M (1968) Vitamin-E-Mangel und chronischer Alkoholismus. Zentralblatt für die gesamte Neurologie und Psychiatrie 192:115–116.

25. Gerhard L (1968) Progressive multifokale Leukencephalopathie. Zentralblatt für allgemeine Pathologie und pathologische Anatomie 110.

26. Heinzler F, Gerhard L, Hensell V (1968) Strahlenschäden des Gehirns. Geometrische Dosisverteilung und Verteilungsmuster der histologischen Veränderungen. Zentralblatt für die gesamte Neurologie und Psychiatrie 192.

27. Bergener M, Gerhard L, Jungklaas H (1968) Genetics in amaurotic idiocy. In: Proceedings of the 12th International Congress of Genetics. Tokyo, S 312.

28. Gerhard L (1968) Atlas des Mittel- und Zwischenhirns des Kaninchens. Springer, Heidelberg.

29. Gerhard L (1968) Morphologische Befunde zur Differentialdiagnose "Senile Demenz" und "Cerebralsklerose" In: Deutsche Gesellschaft für Pathologie (Hrsg) Verhandlungen der Deutschen Gesellschaft für Pathologie, S 164–174.

30. Gerhard L, Schettler G, Strassburg M (1969) Eine foudroyant verlaufende Retikulose mit Erstsymptomen an der Mundschleimhaut. Deutsche Zahnärztliche Zeitschrift 24:210-218.

31. Gerhard L, Olszewski J (1969) Medulla oblongata. In: Hofer H, Schultz A, Starck D (Hrsg) Primatologia, Handbuch der Primatenkunde. Karger, Basel und New York.

32. Hueber R, Gerhard L, Bergener M (1969) Zur bilateralen Nekrose von Ammonshorn und basalen Schläfenlappen. Deutsche Zeitschrift für Nervenheilkunde 196:275–286.

33. Bergener M, Gerhard L, Jungklaas F (1969) Ultrastruktur der Speichersubstanz bei Spätform der amaurotischen Idiotie. In: Deutsche Gesellschaft für Elektronenmikroskopie (Hrsg) Arbeitsgemeinschaft für Ultrastrukturforschung, Wien.

34. Hensell W, Gerhard L, Heinzler F (1969) Strahlenspätschäden des Hirns nach Tumorbestrahlung. Acta Neurochirurgica 20:228–229.

35. Gerhard L, Huchzermayer M, Golonbek M (1969) Zur Morphologie der Myoklonusepilepsie. Zentralblatt für allgemeine Pathologie und pathologische Anatomie 112:611–613.

36. Gerhard L, Huchzermayer M, Golonbek M, Bergener M (1970) Myoklonusepilepsie mit „atypischen“ Myoklonuskörpern. Zentralblatt für die gesamte Neurologie und Psychiatrie 197:339.

37. Bergener M, Gerhard L (1970) Myoklonuskörperkrankheit und progressive Myoklonusepilepsie. Der Nervenarzt 41:166–173.

38. Gerhard L, Huchzermayer H, Debuch H (1970) Histochemische und biologische Befunde bei Lafora'scher Krankheit einer angeborenen Störung des Kohlenhydratstoffwechsels. Hoppe-Seyler's Zeitschrift für Physiologische Chemie 351:1303.

39. Brölsch C, Gerhard L (1970) Traumatische Schädigung des Hippocampus. Zentralblatt für allgemeine Pathologie und pathologische Anatomie 113:244.

40. Gerhard L, Brölsch C (1970) Veränderungen am basalen Schläfenlappen und Gyrus hippocampus beim Schädelhirntrauma. Acta Neuropathologica 15:20–33.

41. Bergener M, Klages W, Flegel H, Gerhard L (1970) Psychopathologie und Motorik bei präseniler und seniler Demenz. Max-Planck-Institut für wissenschaftlichen Film, Göttingen.

42. Gerhard L, Reinhardt V, Solbach H (1970) Zur Morphologie und Ätiologie der Encephalopathie bei Morbus Addison. In: Deutsche Gesellschaft für Pathologie (Hrsg) Verhandlungen der Deutschen Gesellschaft für Pathologie, S 305–312.

43. Gerhard L, Golonbek M (1970) Atypical tumors associated with phacomatoses. 6th International Congress of Neuropathology, Paris, 31.08-05.09.1970, Paris.

44. Gerhard L (1971) Zur Frage der Abgrenzung hypertonischer Encephalopathien von kongophiler Angiopathie bei seniler Demenz und „atypischer“ Alzheimer'scher Krankheit. Verlag der Wiener Akademie der Wissenschaften, Wien.

45. Spancken E, Gerhard L (1971) Spätschädigung des Rückenmarks nach Starkstromunfall. Zentralblatt für allgemeine Pathologie und pathologische Anatomie 114:605.

46. Gerhard L, Reinhardt V, Deutsche Gesellschaft für Pathologie (1971) Morphologische Befunde bei spätinfantiler amaurotischer Idiotie (Typ Bielschowsky). In: Verhandlungen der Deutschen Gesellschaft für Pathologie, S 837.

47. Brölsch C, Gerhard L (1971) Morphologische Befunde zur posttraumatischen Apoplexie. Zentralblatt für allgemeine Pathologie und pathologische Anatomie 114:604–605.

48. Bergener M, Gerhard L (1971) Neurologische Erkrankungen bei chronischem Alkoholismus – Diskussionsbemerkungen zum Übersichtsreferat von Hallen, O., Neundörfer, B. und M. von Rad. Der Nervenarzt 42:437.

49. Gerhard L, Spancken E (1972) Chronische Rückenmarkschädigung nach Starkstromunfall. Acta Neuropathologica 20:357–362.

50. Bergener M, Gerhard L, Husser J (1972) Klinische und morphologische Untersuchungen über eine familiäre Altershalluzinose. Der Nervenarzt 43:18–33.

51. Gerhard L (1972) Neuropathologische Aspekte der gerontopsychiatrischen Terminologie, Meßverfahren und Bewertungsmethoden. In: 1. Symposion der Arbeitsgemeinschaft für Gerontopsychiatrie. Janssen-Symposion, Krefeld, S 72–85.

52. Gerhard L, Bergener M, Homayun S (1972) Angiopathie bei Alzheimer'scher Krankheit. Zeitschrift für Neurologie 201:43–61.

53. Feistkorn R, Gerhard L (1972) Zur Morphologie und Klinik des Psammo-Osteoid-Fibroms der Nasennebenhöhlen. Zentralblatt für allgemeine Pathologie und pathologische Anatomie.

54. Gerhard L, Schmitz-Bauer G (1973) Morphology of cerebral arteries in Marfan's syndrome and medianecrosis idiopathica. Acta Neuropathologica 26:179–184.

55. Gerhard L (1974) Neuropathology of the peripheral visual pathway. Bericht über die Zusammenkunft der Deutschen Ophthalmologischen Gesellschaft 72:8–13.

56. Huchzermeyer H, Gerhard L (1974) Liver in progressive myoclonus epilepsy (Lafora's disease). Klinische Wochenschrift 52:559–567.

57. Mortier W, Michaelis E, Becker J, Gerhard L (1975) Centronuclear myopathy with autosomal dominant inheritance. Humangenetik 27:199–215.

58. Husser J, Gerhard L (1975) The midbrain and psychotic symptoms: findings and problems. Hippokrates 46:418–420.

59. Gerhard L (1976) Neuropathology of forms of reactions. Verhandlungen der Deutschen Gesellschaft für Innere Medizin 82:482–489.

60. Clar HE, Nau, HE, Reinhardt V, Gerhard L (1977) Clinical and morphological studies on development of ventricular widening in adults. Acta Neurochirurgica 36:289-289

61. Clar HE, Gerhard L, Reinhardt V (1978) Experimental and morphological investigations on rabbit concerning reversible and irreversible hypothalamic compression. Acta Neurochirurgica 44:255-255.

62. Schaefer UW, Beyer JH, Boecker WR, Bruntsch U, Gallmeier WM, Hilgard P, Gerhard L et al. (1978) Bone marrow transplantation in acute leukemia. Pathologie Biologie 26:47–48.

63. Weiler G, Reinhardt V, Gerhard L (1978) Distribution patterns and functional relationships of the encephalopathy Wernicke. Zeitschrift für Rechtsmedizin 80:255–258.

64. Bock WJ, Ischebeck W, Gerhard L, Löhr E (1978) Correlation of pathological specimen sections with corresponding computer tomography. Der Radiologe 18:88–91.

65. Clar HE, Reinhardt V, Gerhard L, Hensell V (1979) Clinical and morphological studies of pineal tumours. Acta Neurochirurgica 46:59–76.

66. Bock WJ, Clar HE, Gerhard L, Weichert, HC (1979) Follow-up Studies on Tumors in the posterior fossa with computerized tomogram in children. Acta Neurochirurgica 50:139-140.

67. Nahser C, Nau HE, Hensel D, Gerhard L (1979) Intracranial bleeding in the newborn - clinical and CT considerations. Childs Brain 5:575-575.

68. Nahser C, Flossdorf R, Clar HE, Gerhard L (1980) Development of brain abscesses - computerized tomogram compared with morphological studies. Acta Neurochirurgica 52:130-131.

69. Roosen K, Gerhard L, Schattke HH, Grote W (1980) Radiological and morphological findings after simultaneous cervical interbody fusion in dogs with autogenous bone-grafts and methyl-methacrylate. Acta Neurochirurgica 52:155-155.

70. Clar HE, Andler W, Erbs I, Reinhard V, Gerhard L, Kruczewski F et al. (1980) Experimental hypothalamic dysfunction in dogs. Neurosurgical Review 3:51–55.

71. Weiler G, Reinhardt V, Nau HE, Gerhard L (1980) Contribution of intracranial traumatic aneurysm. Zeitschrift für Rechtsmedizin 85:225–233.

72. Clar HE, Bock WJ, Nahser HC, Gerhard L, Flossdorf R (1980) Computertomographic and morphological findings in cerebral infarctions and intracerebral haematomas in identical sections. Fortschritte auf dem Gebiet der Röntgenstrahlen und der bildgebenden Verfahren 132:118–123.

73. Reinhardt V, Roosen K, Nau HE, Gerhard L (1981) Prognosis of cervical spine lesions. Acta Neurochirurgica 56:273-274.

74. Flossdorf R, Reinhardt V, Gerhard L (1981) Morphological studies in malignant tumors of the peripheral nervous system (neurofibrosarcoma, malignant schwannoma, schwann cell sarcoma). Acta Neuropathologica Supplementum 7:129–133.

75. Nahser HC, Gerhard L, Reinhardt V, Nau HE, Bamberg M (1981) Diffuse and multicentric brain tumors – correlation of histological, clinical and CT appearance. Acta Neuropathologica Supplementum 7:101–104.

76. Nahser HC, Grote W, Löhr E, Gerhard L (1981) Multiple meningiomas. Clinical and computer tomographic observations. Neuroradiology 21:259–263.

77. Bock WJ, Clar HE, Weichert HC, Gerhard L (1981) Follow-up studies in the posterior fossa in children using computerized tomography. Acta Neurochirurgia 24:2–5.

78. Feiden W, Reinhardt V, Gerhard L (1981) Immunofluorescence-microscopy and electron-microscopy studies of vessel amyloidoses of the central nervous-system. Der Pathologe 2:126-127.

79. Nahser HC, Gerhard L (1981) Problem of multicentric and multifocal brain-tumors. Der Pathologe 2:198-196.

80. Schattke HH, Gerhard L, Roosen K, Grote W (1981) Radiological and morphological findings following animal experimental simultaneous spinal body perfusion with bone-cement and auto-transplant. Der Pathologe 3:57-58.

81. Hielscher H, Becker J, Gerhard L (1982) Affection of the nervous system in Behçet's disease – the clinical and neuropathological picture. Fortschritte der Neurologie, Psychiatrie und ihrer Grenzgebiete 50:337–348.

82. Bayindir C, Dambska M, Gerhard L (1982) Distribution pattern of hypoxidosis damage in cases of cardiac-arrest (hypotensive brain-stem necrosis). Der Pathologe 3:115-115.

83. Haan J, Müller E, Gerhard L (1983) Spongiform leukodystrophy following drug abuse. Der Nervenarzt 54:489–490.

84. Mehdorn HM, Wiedemeyer H, Reinhardt V, Gerhard L, Zais E (1983) Histological changes in the superficial temporal artery and their significance for extracranial-intracranial bypass operations. Neurochirurgia 26:181–186.

85. Reinhardt V, Gerhard L, Nau HE, Nahser HC (1983) Craniocerebral injuries in childhood. Neurochirurgia 26:177–180.

86. Vieregge P, Nahser HC, Gerhard L, Reinhardt V, Nau HE (1984) Multiple sclerosis and cerebral tumor. Clinical Neuropathology 3:10–21.

87. Vieregge P, Reinhardt V, Gerhard L, Schliwinski U, Jörg JR (1985) Untreated borderline-leprosy in the ulnar nerve: light and electron microscopical studies. Leprosy Review 56:5–15.

88. Feiden W, Feiden U, Gerhard L, Reinhardt V, Wandeler A (1985) Rabies encephalitis: immunohistochemical investigations. Clinical Neuropathology 4:156–164.

89. Feiden W, Gerhard L, Borchard F (1986) Neuritis cordis in acute Guillain-Barré polyneuritis. Deutsche Medizinische Wochenschrift 111:1382.

90. Nau HE, Gerhard L, Foerster M, Nahser HC, Reinhardt V, Joka T (1987) Optic nerve trauma: clinical, electrophysiological and histological remarks. Acta Neurochirurgica 89:16–27.

91. Vieregge P, Gerhard L, Nahser HC (1987) Familial glioma: occurrence within the familial cancer syndrome and systemic malformations. Journal of Neurology 234:220–232.

92. Feiden W, Gerhard L, Borchard F (1988) Neuritis cordis due to the acute polyneuritis of the Guillain-Barré syndrome. Virchows Archiv A – Pathologische Anatomie und Histopathologie 413:573–580.

93. Bamberg M, Budach V, Stuschke M, Gerhard L, Streffer C (1988) Heterotransplantation of a human glioma and brain metastases in the athymic nude mouse – a preclinical model for radiation oncology. Strahlentherapie und Onkologie 164:235–243.

94. Bamberg M, Budach V, Stuschke M, Gerhard L (1988) Preliminary experimental results with the nitrosourea derivative ACNU in the treatment of malignant gliomas. Radiotherapy and Oncology 12:25–29.

95. Feiden W, Kaiser E, Gerhard L, Dahme E, Gylstorff B, Wandeler A et al. (1988) Immunohistochemical staining of rabies virus antigen with monoclonal and polyclonal antibodies in paraffin tissue sections. Zentralblatt für Veterinärmedizin Reihe B 35:247–255.

96. Wiedemayer H, Roosen K, Kalff R, von Frankenberg S, Gerhard L (1988) Therapy and prognosis of brain tumors in the first years of life. Neurochirurgia 1:196–198.

97. Vieregge P, Gerhard L, Reinhardt V (1988) Intracranial space-occupying processes in psychiatry – 30 years of clinico-neuropathologic catamnesis. Fortschritte der Neurologie, Psychiatrie und ihrer Grenzgebiete 56:373–379.

98. Wiedemayer H, Nau HE, Reinhardt V, Gerhard L, Grote W (1989) Syringomyelia from the neurosurgical viewpoint. Der Nervenarzt 60:17–25.

99. Stuschke M, Bamberg M, Budach V, Gerhard L, Sack H (1989) Dose response relationships in two xenografted human gliomas after fractionated radiotherapy. Strahlentherapie und Onkologie 165:516–517.

100. Kilian F, Nau HE, Langer C, Wiedemayer H, Reinhardt V, Gerhard L (1990) Death in a neurosurgical intensive care unit. Analysis of 109 deceased intensive care patients. Wiener Medizinische Wochenschrift 140:559–561.

101. Grabbe HD, Haan J, Brandt J, Gerhard L (1990) Traumatic rupture of the inferior petrous sinus with fatal subarachnoid hemorrhage after a fall on the back of the head. Der Nervenarzt 61:504–506.

102. Gerhard L, Hugo HH (1991) Mycoses of the central nervous system. Mycoses 1:21–24.

103. Patt S, Gertz HJ, Gerhard L, Cervós-Navarro J (1991) Pathological changes in dendrites of substantia nigra neurons in Parkinson's disease: a Golgi study. Histology and Histopathology 6:373–380.

104. Mehdorn HM, Gerhard L, Müller SP, Olbrich HM (1992) Clinical and cerebral blood flow studies in patients with intracranial hemorrhage and amyloid angiopathy typical of Alzheimer's disease. Neurosurgical Review 15:111–116.

105. Feldges A, Gerhard L, Reinhardt V, Budach V (1992) Primary cerebral anaplastic T-cell lymphoma (type Ki-1): review and case report. Clinical Neuropathology 11:55–59.

106. Linke RP, Gerhard L, Lottspeich F (1992) Brain-restricted amyloidoma of immunoglobulin lambda-light chain origin clinically resembling multiple sclerosis. Biological Chemistry Hoppe-Seyler 373:1201–1209.

107. Patt S, Gerhard L (1993) A Golgi study of human locus coeruleus in normal brains and in Parkinson's disease. Neuropathology and Applied Neurobiology 19:519–523.

108. Wiedemayer H, Nau HE, Rauhut F, Grote W, Gerhard L (1994) Operative treatment and prognosis of syringomyelia. Neurosurgical Review 17:37–41.

109. Patt S, Gerhard L, Zill E (1994) A Golgi study on the red nucleus in man. Histology and Histopathology 9:7–10.

110. Bergmann M, Terzija-Wessel U, Blasius S, Kuchelmeister K, Kryne-Kubat B, Gerhard L et al. (1994) Intravascular lymphomatosis of the CNS: clinicopathologic study and search for expression of oncoproteins and Epstein-Barr virus. Clinical Neurology and Neurosurgery 96:236–243.

111. Wang Z, Gerhard L (1995) Reexpression of nerve growth factor receptor in human traumatic injured spinal cord. Chinese Medical Journal (English Edition) 108:438–443.

112. Heinz A, Schmidt LG, Winterer G, Gerhard L, Przuntek H (1995) Optical and tactile hallucinosis as clinical onset of Creutzfeldt-Jakob disease. Der Nervenarzt 66:712–716.

113. Wang ZH, Walter GF, Gerhard L (1996) The expression of nerve growth factor receptor on Schwann cells and the effect of these cells on the regeneration of axons in traumatically injured human spinal cord. Acta Neuropathologica 91:180–184.

114. Goebel HH, Gerhard L, Kominami E, Haltia M (1996) Neuronal ceroid-lipofuscinosis – late-infantile or Jansky-Bielschowsky type revisited. Brain Pathology 6:225–228.

115. Wheeler RB, Schlie M, Kominami E, Gerhard L, Goebel HH (2001) Neuronal ceroid lipofuscinosis: late infantile or Jansky-Bielschowsky type revisited. Acta Neuropathologica 102:485–488.

116. Davis RL, Shrimpton AE, Carrell RW, Lomas DA, Gerhard L, Baumann B et al. (2002) Association between conformational mutations in neuroserpin and onset and severity of dementia. The Lancet 359:2242–2247.

117. Bielau H, Trübner K, Krell D, Agelink MW, Bernstein HG, Stauch R et al. (2005) Volume deficits of subcortical nuclei in mood disorders – a postmortem study. European Archives of Psychiatry and Clinical Neuroscience 255:401–412.
